# Investigation on the Controllable Synthesis of Colorized and Magnetic Polystyrene Beads With Millimeter Size *via In Situ* Suspension Polymerization

**DOI:** 10.3389/fchem.2022.891582

**Published:** 2022-05-27

**Authors:** Juntao Yan, Hua Wu, Pan Huang, Yourong Wang, Bowang Shu, Xiaofang Li, Deng Ding, Ya Sun, Chunlei Wang, Jian Wu, Linbing Sun

**Affiliations:** ^1^ College of Chemistry and Environmental Engineering, Wuhan Polytechnic University, Wuhan, China; ^2^ College of Advanced Interdisciplinary Studies, National University of Defense Technology, Changsha, China; ^3^ College of Chemical Engineering, Nanjing Tech University, Nanjing, China

**Keywords:** colorized polystyrene bead, Fe_3_O_4_, micrometer size, controllable synthesis, *in situ* suspension polymerization

## Abstract

A series of colorized and magnetic polystyrene/Fe_3_O_4_ (PS/Fe_3_O_4_) composite beads with millimeter size are successfully synthesized by introducing hydrophobic Fe_3_O_4_ *via in situ* suspension polymerization of styrene for the first time. Effects of the hydrophobic Fe_3_O_4_ content, stirring speed, and surfactant dosage on the macromorphology and particle size of PS/Fe_3_O_4_ beads are systematically investigated to realize the controllable synthesis. Moreover, three kinds of hydrophobic pigments are also employed to synthesize colorized polystyrene, which demonstrates the versatility, simplicity, and wide applicability of the proposed method. Scanning electron microscopy (SEM) and element mapping (EM) images demonstrated that the hydrophobic Fe_3_O_4_ is well dispersed in the polystyrene matrix. Thermogravimetric analysis (TGA) shows that the resultant PS/Fe_3_O_4_ beads possess a better thermal stability than neat PS. PS/Fe_3_O_4_ beads have a promising application in the fields of colorized extruded PS board, colorized expanded PS foam particle, and board.

## Introduction

As is well known that polystyrene (PS) is one of the most common plastics in the modern chemical industry, and expanded polystyrene (EPS) foam is widely used in building insulation, packaging materials, sound insulation materials, decorative materials, and other fields (Xing et al., 2013; Sun and Lawrence, 2017; Huang et al., 2018; Ito et al., 2018; Wang et al., 2018; Peng et al., 2020; Hamann et al., 2021; Cao et al., 2022; Liu and Peng, 2022). With the increasing competition of PS and EPS markets at home and abroad, requirements for the performance and function of PS and EPS are put forward in the above-mentioned fields. There are many researchers paying attention to the fabrication of multifunctional PS and EPS.

Generally speaking, many inorganic fillers are incorporated into the PS matrix to fabricate PS composites, which are expected to possess combined properties of both inorganic filler and PS; the inorganic filler can impart the PS with unique properties, such as electrical, mechanical, magnetic, colorful, thermal, flame retarding, and so on. Many PS/inorganic hybrids with improved properties have been synthesized by different methods. For example, Zhou et al. (2016) have synthesized graphene-based smoke suppression agents by a solvothermal method for improving the flame retardation of polymer composites, and the graphene-based smoke suppression agent is incorporated into the PS matrix by a masterbatch melt blending method to enhance the thermal stability and flame retardance of PS. Chen et al. (2003) have synthesized the conductive PS/graphite nanosheet nanocomposite films *via in situ* polymerization of monomer in the presence of sonicated expanded graphite during sonication. Nyambo et al. (2008) have achieved the flame-retarded PS by incorporating the layered double hydroxides and ammonium polyphosphate *via* the melt blending method. Yang et al. (2014) have prepared PS/attapulgite nanocomposites *via in situ* suspension polymerization in the presence of functionalized attapulgite with the redox initiation system; the mechanical properties and thermal stability of PS/attapulgite nanocomposites are effectively improved due to the stronger interfacial bonding. Faraguna et al. (2017) have achieved PS nanocomposites with functionalized carbon nanotubes by the melt and solution mixing process, and they exhibited an in-depth investigation on the dispersion, melt rheology, and electrical and thermal properties of PS-MWCNT nanocomposites, which are prepared by the melt and solution mixing method. Azizi et al. (2019) have synthesized PS-modified GO using an ammonium persulfate initiator, which is used as an adsorbent for the removal of three anionic dyes from wastewater. Nikpour et al. (2022) have fabricated PS/Fe-MOF composite beads by a two-step method consisting of the synthesis of Fe-MOF and filling Fe-MOF into the PS matrix by the phase inversion method; the achieved material is utilized to uptake and separate various pollutions. Hong et al. (2009) have fabricated PS/Fe_3_O_4_ composite particles with a diameter of 200 nm *via* inverse emulsion polymerization. Liu et al. (2011) have obtained monodisperse magnetic PS/Fe_3_O_4_ microspheres with a mean size of 1078 nm, which is prepared by the soap-free emulsion polymerization and magnetic colloid swelling polymerization. Naeemikhah et al. (2019) have synthesized the magnetic crosslinked PS with hydrophilic nature by the solvent-free surface-initiated atom transfer radical polymerization method, and the organically modified Fe_3_O_4_ is used as the polyaddition initiator.

Many studies regarding functional PS composites have been reported (Yan et al., 2011a; Yan et al., 2011b; Zheng et al., 2013; Umar et al., 2014; He et al., 2015; Wang et al., 2018; An et al., 2019; Li et al., 2019; Shi et al., 2021; Wang et al., 2022a; Wang et al., 2022b); moreover, PS/Fe_3_O_4_ nanomaterials and micrometer size composites have been synthesized in the above studies; however, there is no report regarding the colorized and magnetic PS/Fe_3_O_4_ beads with millimeter size. To the best of our knowledge regarding functional PS, many colorful extruding polystyrenes (XPS) are achieved by blending the PS with organic or inorganic pigments. However, due to the instability and flammability of the organic pigment, its application in PS is therefore restricted. In addition, the blending method results in poor mixing owing to the interfacial incompatibility between PS beads and inorganic pigments. Therefore, an effective method aimed at solving the above disadvantages is of great necessity.

Black pigment Fe_3_O_4_ (iron black) particles have widespread applications in the coating industry, coloration, magnetic bioseparation, drug delivery, targeted drug, toner preparation, and construction industry due to their excellent properties of alkali resistance, black color, magnetic properties, nontoxicity, superparamagnetism, high saturation magnetization, high magnetic susceptibility, and biocompatibility (Wang et al., 2011; Wang et al., 2013; Yu et al., 2017; Rahimi et al., 2020; Fang et al., 2021; Hou et al., 2022; Wang et al., 2022a). Moreover, Fe_3_O_4_ particles are naturally hydrophilic due to the abundant hydroxyl groups on the particle surface, and the hydrophobic Fe_3_O_4_ can be easily obtained by the surface modification to improve its interfacial compatibility with the monomer and polymer matrices.

In the present study, the oleic acid-modified Fe_3_O_4_ (OA-Fe_3_O_4_) particles with hydrophobic features are introduced into the PS matrix *via* the *in situ* suspension polymerization of styrene, by which the magnetic and color properties of Fe_3_O_4_ are imparted to PS/Fe_3_O_4_ composite beads. Different Fe_3_O_4_ contents and color degrees of PS/Fe_3_O_4_ composite beads with millimeter size can be obtained by regulating the Fe_3_O_4_ dosage. Effects of the OA-Fe_3_O_4_ dosage, surfactant dosage and stirring speed on the macromorphology, particle size, and size distribution of PS/Fe_3_O_4_ composite beads are systematically investigated to realize the controllable synthesis, and the optimum process conditions are determined. Scanning electron microscopy (SEM) and element mapping (EM) images demonstrate that the modified Fe_3_O_4_ is well dispersed in the PS/Fe_3_O_4_ composite beads; thermogravimetric analysis (TGA) shows that the resultant PS/Fe_3_O_4_ composite beads possess a better thermal stability than bare PS. The novelty of this article lies in the synthesis of colorized and magnetic PS/Fe_3_O_4_ beads with millimeter size by the *in situ* suspension polymerization technique, which may bring about a promising future in the large-scale production of colorful and magnetic PS/Fe_3_O_4_ composite beads or EPS foam due to the simplicity and versatility of the synthetic process.

## Experimental Section

### Materials

Styrene (St, 99%, Shanghai Chemical Reagent Company, China) was distilled under a nitrogen atmosphere and reduced pressure prior to polymerization. Benzoyl peroxide (BPO, Sinopharm Chemical Reagent Co., Ltd., China) was purified by recrystallization before usage. Calcium-trihydroxy phosphate, tertiary calcium phosphate (TCP), sodium dodecylbenzene sulfonate (SDBS), and sodium sulfate (Na_2_SO_4_) were used as received. Oleic acid (OA, Tianjin Guangfu Chemical Reagents Company) and pentane (Sinopharm Chemical Reagents Company) were the analytical reagents. PVA with a polymerization degree of 1750 ± 50 was supplied by Sinopharm Chemical Reagents Company. The water used in this experiment was distilled followed by deionization. In addition, Fe_3_O_4_ (20 nm, 99%) was supplied by Nanjing AiPuRui Nano Material Co., Ltd. Three kinds of iron oxides, red, dark green, and China blue pigments, were supplied by Zhejiang Taizhu Group Co., Ltd.

### Surface Modification of Fe_3_O_4_ Magnetic Nanoparticles

The hydrophobic Fe_3_O_4_ could be achieved by a facile water phase modification process. Firstly, 4 g of Fe_3_O_4_ nanoparticles and 150 ml of deionized water were put into a 250 ml three-neck flask equipped with a mechanical stirrer; the suspension was fully stirred at 400 rpm for 20 min, and then a certain amount of OA that used as a modifying agent was charged into the mixture system; thereafter, the flask was placed into a water bath at a constant temperature of 80°C for 1 h. The product was obtained by a magnet and was washed by deionized water and EtOH continuously for two times to remove the physically adsorbed superfluous oleic acid, after which the black precipitate was dispersed in cyclohexane and dried by nitrogen gas. Then, the OA-modified Fe_3_O_4_ with a strong hydrophobic surface was achieved and was denoted by OA-Fe_3_O_4_, which could significantly ameliorate the compatibility between inorganic particles and styrene monomer. Hydrophobic Fe_2_O_3_, dark green, and China blue inorganic pigments were also modified similar to the above procedure.

### Synthesis of PS/Fe_3_O_4_ Beads *via In Situ* Suspension Polymerization

**FIGURE 1:**
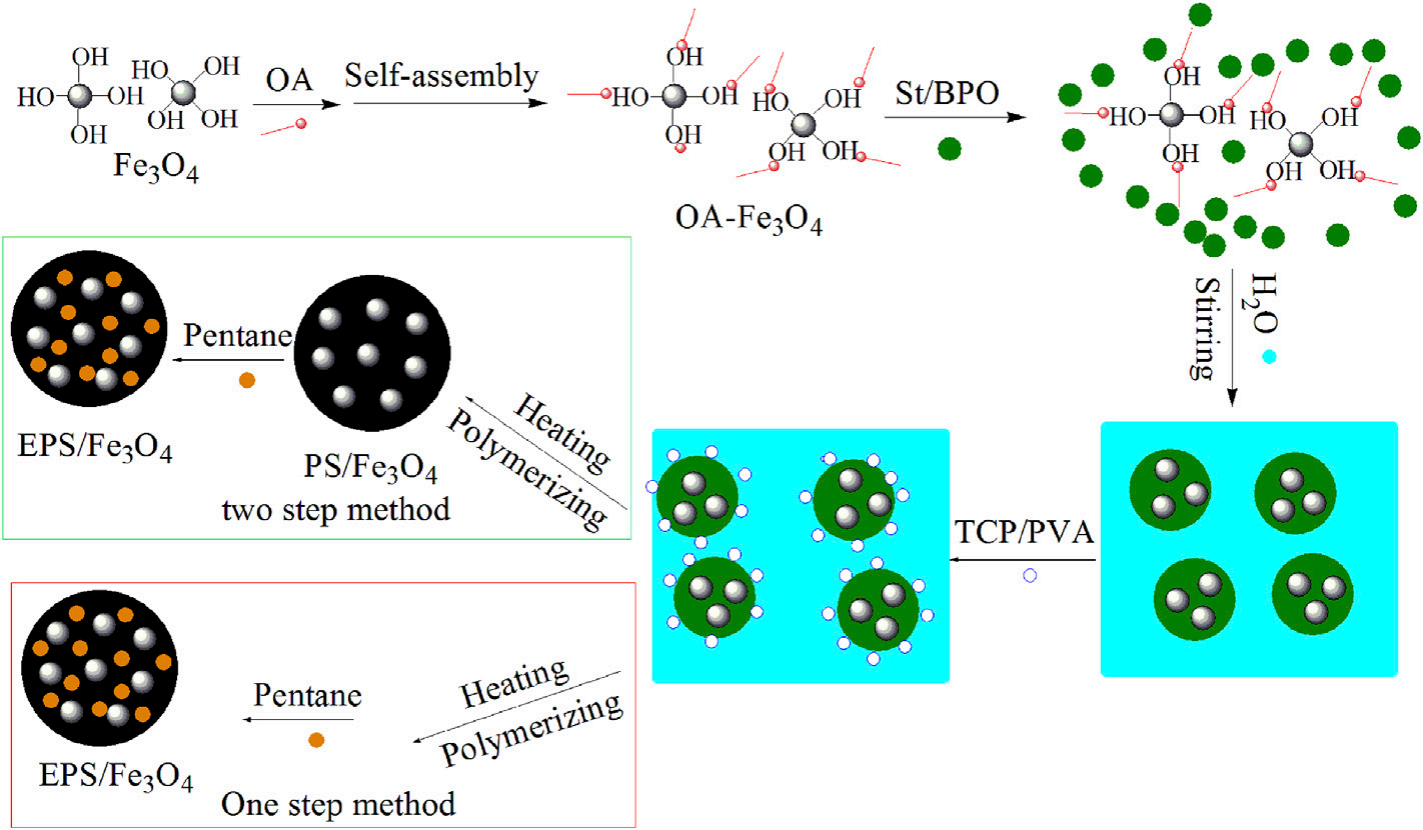
Schematic showing the synthesis of colorized PS/Fe_3_O_4_ beads and EPS/Fe_3_O_4_ beads.

The colorized PS/Fe_3_O_4_ beads were successfully synthesized *via in situ* suspension polymerization of styrene in the presence of OA-Fe_3_O_4_, using BPO as an initiator, PVA as organic stabilizers, and TCP and Na_2_SO_4_ as inorganic stabilizers. The complex stabilizers of TCP and PVA were used to hinder the coalescence and break-up of droplets during the suspension polymerization in this article. SDBS aqueous solution was used to reduce the surface tension of the suspending medium H_2_O and improve the stabilization of the inorganic stabilizer at the interface of styrene and H_2_O. The overall schematic procedure used to synthesize the colorized PS/Fe_3_O_4_ beads and EPS/Fe_3_O_4_ beads is illustrated in Figure 1; the detailed recipes and relevant physical parameters of the PS/Fe_3_O_4_ beads are summarized in Table 1, and the synthesis procedure was described as follows: as-prepared PS/Fe_3_O_4_ beads were immersed in a blowing agent pentane to obtain the expandable polystyrene/Fe_3_O_4_ (EPS/Fe_3_O_4_) beads by means of a two-step method. Moreover, EPS/Fe_3_O_4_ beads can also be directly obtained when the blowing agent pentane was impregnated during *in situ* suspension polymerization of styrene by means of a one-step method, and these two methods will be discussed in detail in our future research.

**TABLE 1:** Recipes of the synthesis of PS/Fe_3_O_4_ composite beads.

Sample No.	1	2	3	4	5	6	7	8	9
H_2_O (ml)	80	80	80	80	80	80	80	80	70
SDBS solution (ml)	10	10	10	10	10	10	10	10	20
PVA solution (ml)	10	10	10	10	10	10	10	10	10
TCP (g)	0.14	0.14	0.14	0.14	0.14	0.14	0.14	0.14	0.14
Na_2_SO_4_ (g)	0.30	0.30	0.30	0.30	0.30	0.30	0.30	0.30	0.30
BPO (g)	0.24	0.24	0.24	0.24	0.24	0.24	0.24	0.24	0.24
St (g)	20	20	20	20	20	20	20	20	20
OA-Fe_3_O_4_ content (%)	0.4	0.8	1.2	1.6	2.0	2.0	2.0	2.0	2.0
Stirring speed (rpm)	180	180	180	180	180	210	240	270	270
Supplemental TCP (g)	0.4	0.4	0.4	0.4	0.4	0.4	0.4	0.4	0.4

To a 250 ml four-necked round-bottom flask fitted with a mechanical overhead stirrer and a condenser, 80 ml deionized water, 0.14 g TCP, 0.30 g Na_2_SO_4_, 10 ml of 0.02 wt% SDBS aqueous solution, and 10 ml of 4 wt% PVA aqueous solution were charged one by one. The above mixture was stirred at 200 rpm to activate the dispersant. In the following procedure, different dosages of OA-Fe_3_O_4_ were dispersed in the styrene and initiator BPO solution (0.24 g BPO was dissolved in 20 g styrene monomer) to form a styrene-based magnetic fluid, and then the styrene-based magnetic fluid was transferred into the above flask. The stirrer was fixed at a certain speed, and the reaction system was heated to 85°C; one portion of 0.2 g TCP was supplemented when the temperature reached 85°C and the other portion of 0.2 g TCP was supplemented after 1 h to prevent the polymerized PS/Fe_3_O_4_ beads from agglomeration with the increasing viscosity. The polymerization reaction was terminated when the colorized PS/Fe_3_O_4_ beads became hardened by sampling from the flask. Finally, the colorized PS/Fe_3_O_4_ beads were washed by dilute acid solution and water to remove the dispersant on the surface of PS/Fe_3_O_4_ beads.

## Characterization

### Static Contact Angle

Static contact angles of water on the powder-pressed pellets were measured with a JC2000C2 contact angle goniometer (Shanghai Zhongchen Powereach Company, China) by the sessile drop method using a microsyringe at 25°C. More contact angles were averaged to get a reliable value for each sample.

### Dynamic Light Scattering

The particle sizes and their distribution of Fe_3_O_4_ and OA-Fe_3_O_4_ were measured by dynamic light scattering (DLS) with a Malven zetasizer 3000 HSA particle sizer.

### X-Ray Diffraction

X-ray diffraction (XRD) data were collected on a Rigaku D/MAX 2550 diffractometer with Cu Ka radiation.

### Fourier Transform Infrared

Fourier transform infrared (FTIR) spectra of KBr powder-pressed pellets were recorded on a Nicolet Instruments Research Series 5PC Fourier transform infrared spectrometer.

### Scanning Electron Microscope

The PS/Fe_3_O_4_ beads were broken at low temperature by liquid nitrogen to ensure a brittle fracture. The cross section was sprayed with gold to release the polymer surface charge for increased contrast. The cross-sectional morphology was imaged on a TESCAN MAIA 3 LMH scanning electron microscope (SEM) to examine the micro-structure. The accelerated voltage was 15 kV.

### Energy-Dispersive Spectrometer and Element Mapping

Energy-dispersive spectrometer (EDS) and EM are attached to the SEM apparatus, which were employed to examine the dispersity of the modified Fe_3_O_4_ in PS/Fe_3_O_4_ beads.

### Thermogravimetric Analysis

TGA was performed with a Pyris 1TGA (Perkin Elmer) under a nitrogen atmosphere at a heating rate of 10°C/min from 25 to 600°C. PS/Fe_3_O_4_ beads were milled into powder, and the sample weight was about 10 mg.

## Results and Discussion

### Effect of OA Dosage on the Hydrophobicity of OA-Fe_3_O_4_

**FIGURE 2:**
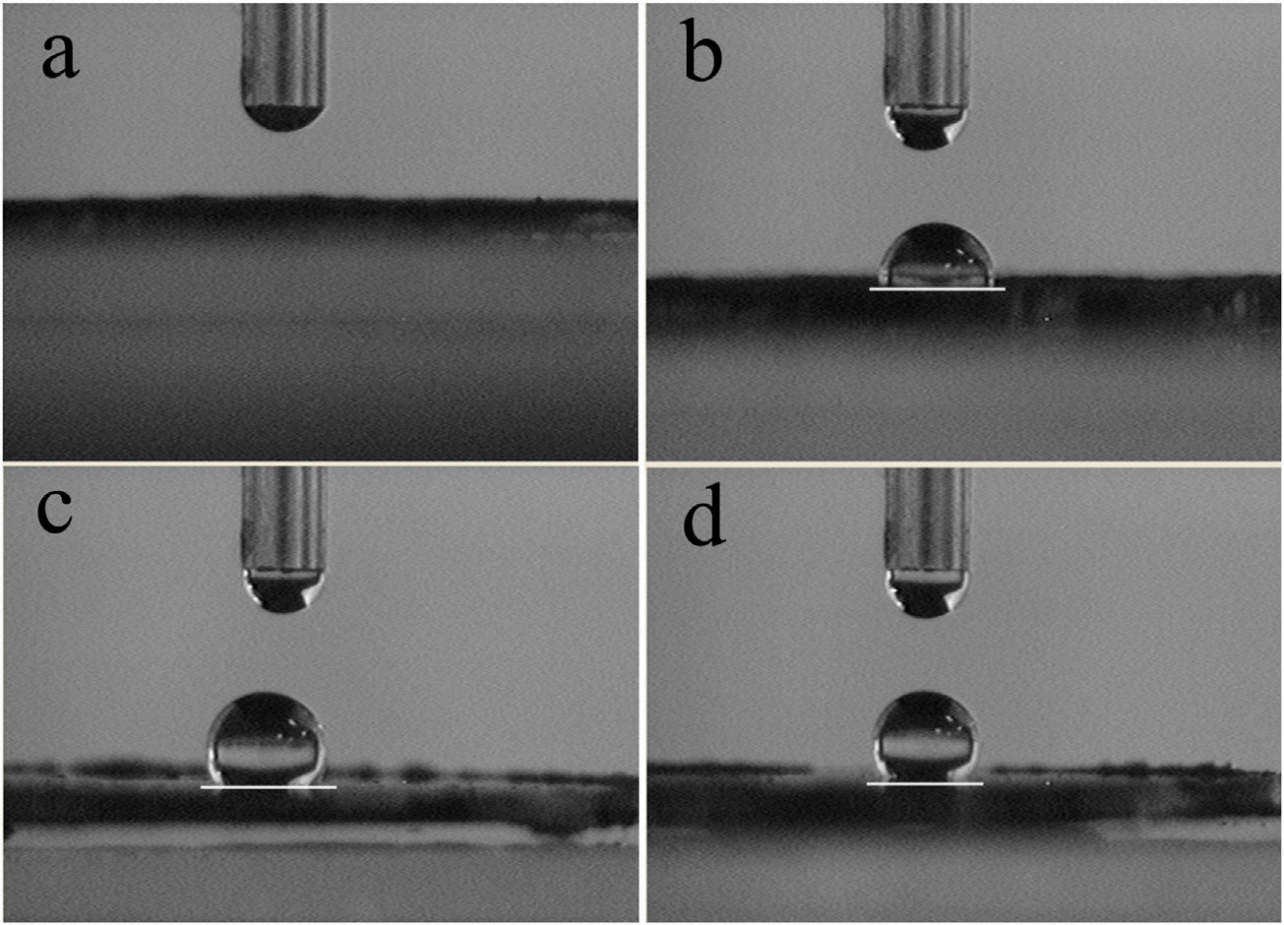
Water contact angle of Fe_3_O_4_ modified by OA with different dosages: **(A)** 0, **(B)** 0.5 wt%, **(C)** 1 wt%, and **(D)** 1.5 wt%.

Static water contact angle test was employed to evaluate the effect of OA dosage (relative to the Fe_3_O_4_ mass) on the surface property of Fe_3_O_4_, as well as to determine the optimum modifier dosage. As seen from Figure 2, when the water droplet was dropped onto a thin pellet of Fe_3_O_4_ without modification, as shown in Figure 2A, no stable water droplet was formed and the water was imbibed in the pellet, while the static contact angle of the OA-Fe_3_O_4_ increased with the increase of OA dosage, and the static contact angles of OA-Fe_3_O_4_ samples in Figures 2B–D were about 92.49°, 122.5°, and 131.68°, respectively. And the results of the static contact angle test indicated that the surface property of Fe_3_O_4_ was changed from hydrophilic to hydrophobic after the modification by OA molecules, which facilitated the dispersion of OA-Fe_3_O_4_ in the styrene monomer due to the improved compatibility. Considering the unobvious change of the static contact angle for the OA dosage of 1 wt% and 1.5 wt%, thus the OA dosage of 1 wt% was determined.

### Particle Size Analysis of Unmodified Fe_3_O_4_ and OA-Fe_3_O_4_

**FIGURE 3:**
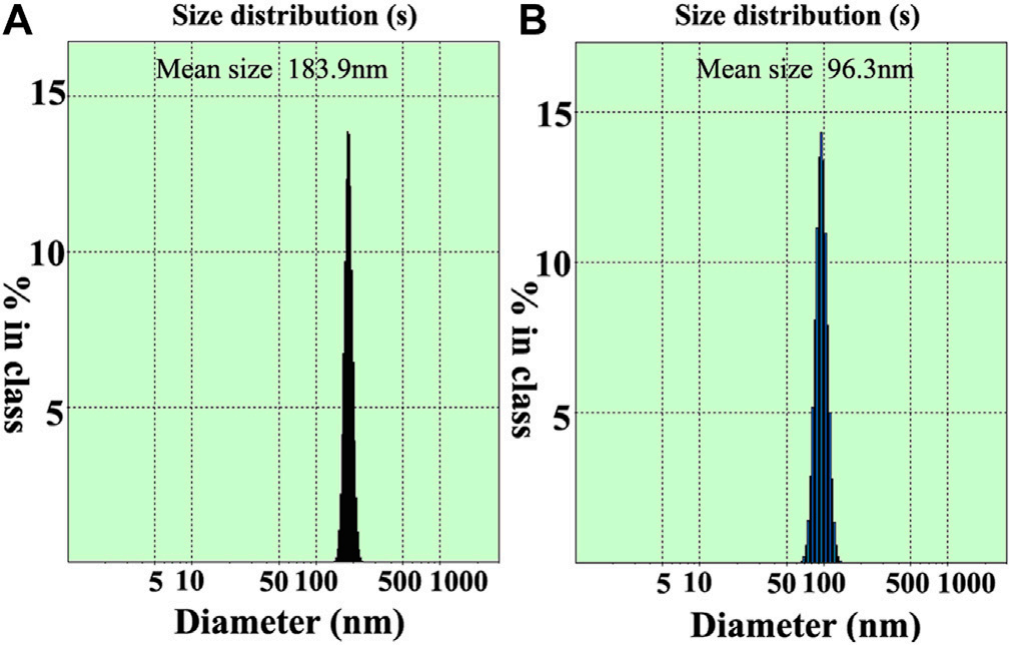
Particle size and its distribution: **(A)** unmodified Fe_3_O_4_ and **(B)** OA-Fe_3_O_4_.

**FIGURE 4:**
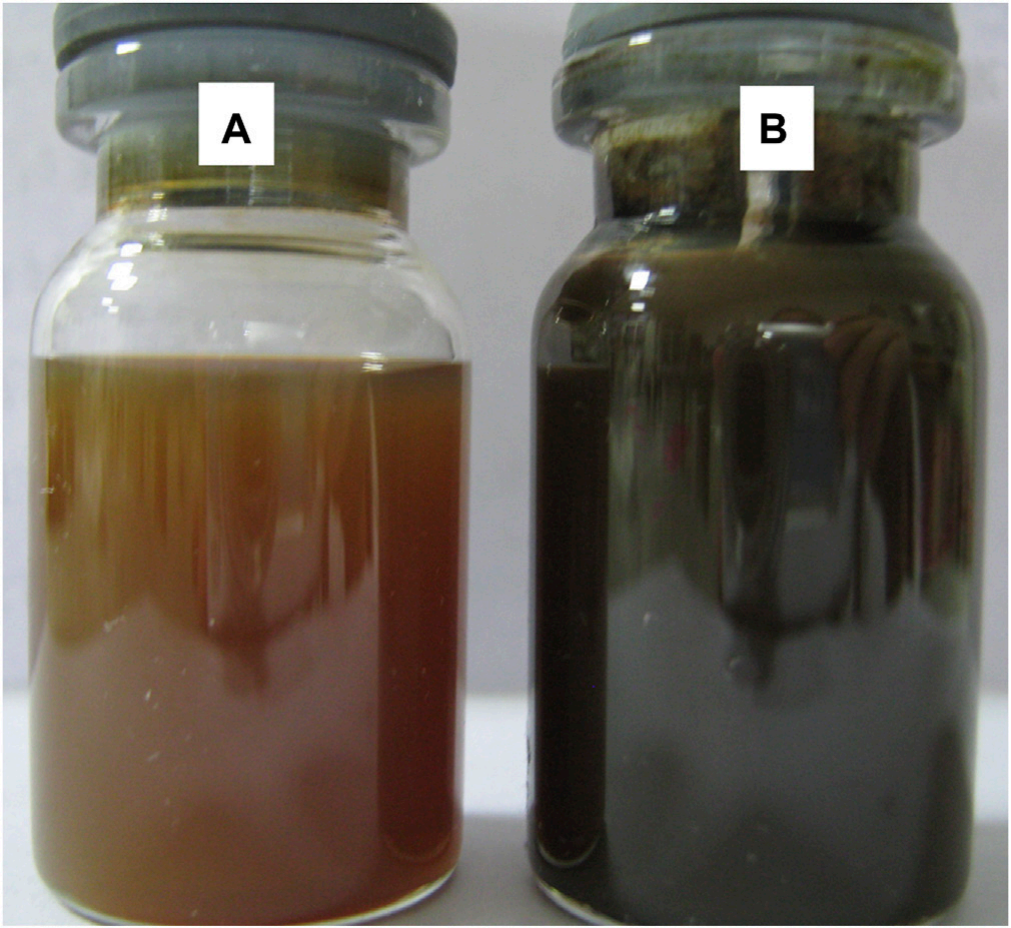
Optical photography of OA-Fe_3_O_4_ dispersed in styrene monomer with different contents: **(A)** 0.8 wt% and **(B)** 1.6 wt%.

In order to examine the modification effect of Fe_3_O_4_, DLS measurement was utilized to provide direct evidence. The same weights of Fe_3_O_4_ and OA-Fe_3_O_4_ were dispersed in H_2_O and styrene monomer, respectively. The particle size and its distribution of Fe_3_O_4_ and OA-Fe_3_O_4_ are shown in Figure 3. The mean sizes of Fe_3_O_4_ and OA-Fe_3_O_4_ were 183.9 and 96.3 nm, and their size distribution widths were 33.7 and 25.6, respectively. It could be seen that both the average particle size and its distribution width distinctly decreased after the modification, which suggested that OA-Fe_3_O_4_ possessed a good interfacial compatibility with styrene monomer, and the particle agglomeration was weakened. Figure 4 shows the optical photography of OA-Fe_3_O_4_ with different contents dispersed in styrene monomer after 1 month, which demonstrated that the OA-Fe_3_O_4_ possessed a good dispersed stability in styrene monomer; moreover, interestingly, the different color of styrene could be achieved by tuning the OA-Fe_3_O_4_ dosage. The above two aspects ensured that the OA-Fe_3_O_4_ particles were well dispersed in the PS matrix by *in situ* suspension polymerization.

### Effect of OA-Fe_3_O_4_ Content on the Macromorphology and Particle Size of PS/Fe_3_O_4_ Beads

**FIGURE 5:**
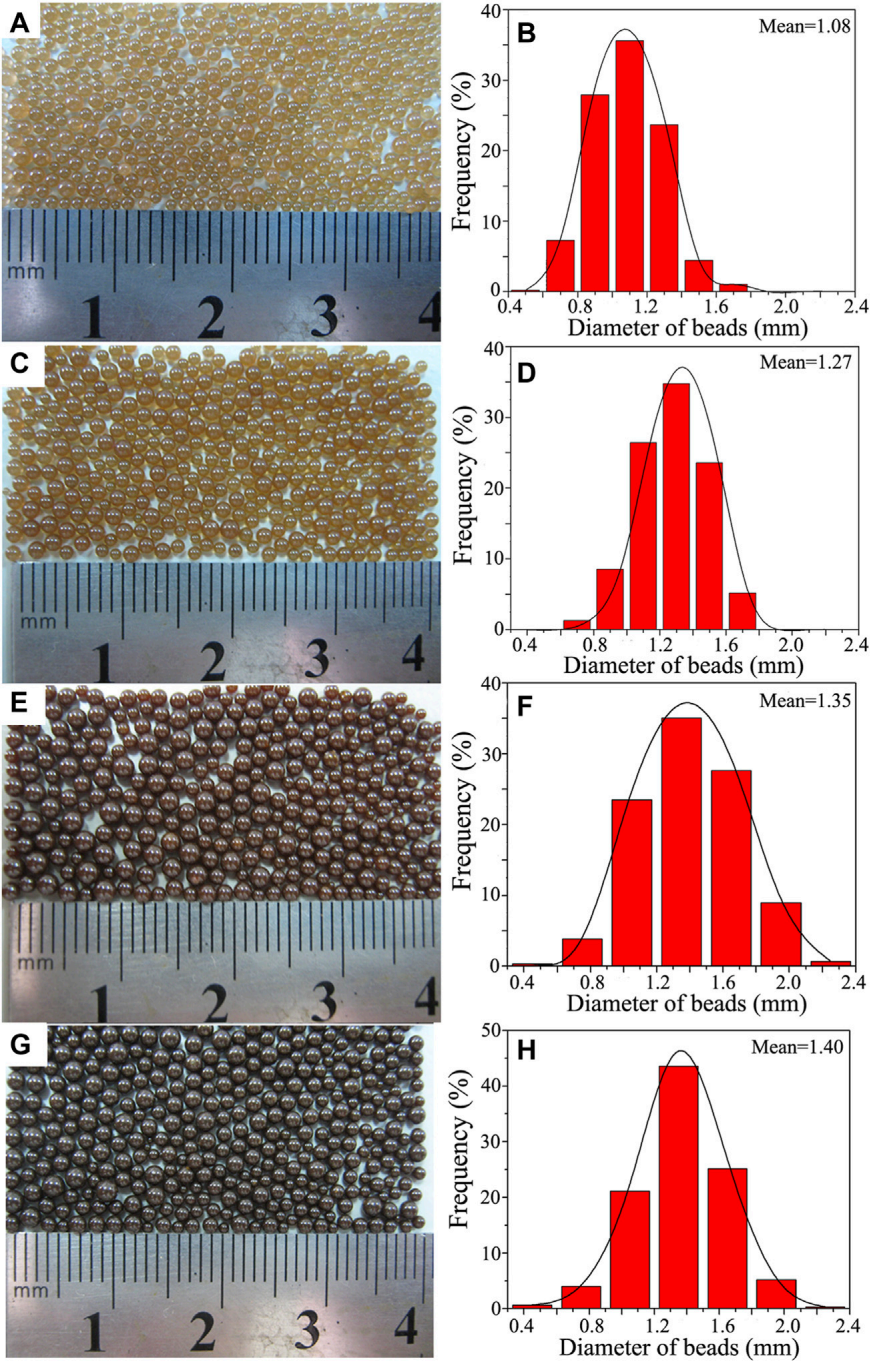
Optical photography showing the macromorphology of PS/Fe_3_O_4_ composite beads obtained with different contents of OA-Fe_3_O_4_: **(A)** 0.4 wt%, **(C)** 0.8 wt%, **(E)** 1.2 wt%, and **(G)** 1.6 wt%. Panels **(B)**, **(D)**, **(F),** and **(H)** are the statistical histograms showing the size distribution of panels **(A)**, **(C)**, **(E),** and **(G)**, respectively.

Uniform particle size and narrow size distribution were expected for the industrial large-scale production of expandable PS. It is because of the fact that a relatively wide size distribution might require a repeated size classification procedure, which is high in cost, time-consuming, and resulted in poor production efficiency. Therefore, the effect of the OA-Fe_3_O_4_ content on the macromorphology, particle size, and size distribution of PS/Fe_3_O_4_ beads was systematically investigated. As seen from optical images of the PS/Fe_3_O_4_ composite beads in Figure 5, a series of PS/Fe_3_O_4_ composite beads with a smooth surface and spherical appearance were successfully synthesized by varying the OA-Fe_3_O_4_ content at the same synthetic condition. First, the color degree of PS/Fe_3_O_4_ composite beads became deeper with the increase of the OA-Fe_3_O_4_ content. When the OA-Fe_3_O_4_ content was 0.4 wt%, light yellow PS/Fe_3_O_4_ beads in Figure 5A were achieved. When the OA-Fe_3_O_4_ content increased from 0.8 to 1.2 and to 1.6 wt%, yellow, brown, and dark brown PS/Fe_3_O_4_ beads were obtained in Figures 5B–D, respectively. Thus, PS/Fe_3_O_4_ beads with different color degrees could be realized by tuning the OA-Fe_3_O_4_ content, which could be fabricated into various color PS products.

ImageJ software was employed to measure the diameter of all the PS/Fe_3_O_4_ beads in Figures 5A,C,E,G; the statistical histogram results of the corresponding samples are presented in Figures 5B,D,F,H, respectively. And the detailed results are listed in Table 2. It has been noted that the particle size distribution of PS/Fe_3_O_4_ beads in Figure 5A mainly concentrated in the range of 0.9–1.3 mm, accounting for 87.08%, and its mean size was 1.08 mm. The particle size distribution of PS/Fe_3_O_4_ beads in Figure 5C mainly concentrated in the range of 1.1–1.5 mm, accounting for 84.72%, and its mean size was 1.27 mm. The particle size distribution of PS/Fe_3_O_4_ beads in Figure 5E mainly concentrated in the range of 1.05–1.65 mm, accounting for 86.17%, and its mean size was 1.35 mm. The particle size distribution of PS/Fe_3_O_4_ beads in Figure 5G mainly concentrated in the range of 1.05–1.65 mm, accounting for 89.88%, and its mean size was 1.40 mm. Based on the above results, it has been found that the particle size distribution became wider with the increase in the OA-Fe_3_O_4_ content, and the mean size became larger as well. This phenomenon could be explained as follows: on the one hand, it is known that polymerization reaction occurred within the monomer droplet for the suspension polymerization of styrene, and the viscosity of the polymerized monomer droplet increased as the monomer conversion increased during the polymerization reaction, especially during the auto-acceleration stage of polymerization reaction (Yan et al., 2011a); thus, the coalescence possibility of the polymerized monomer droplet increased as well. On the other hand, for every polymerized monomer droplet unit containing OA-Fe_3_O_4_ nanoparticles, the increasing OA-Fe_3_O_4_ content also resulted in the viscosity increment of the polymerized monomer droplet, because the structural formula of oleic acid was CH_3_(CH_2_)_7_CH = CH(CH_2_)_7_COOH, and there was a “-CH = CH-” double bond in the structural formula; the “-CH = CH-” double bond took part in the polymerization reaction of styrene monomer, which might accelerate the polymerization rate of a monomer.

**TABLE 2:** Size distribution of PS/Fe_3_O_4_ composite beads.

Sample No.	Size distribution (mm)	Frequency (%)	Mean size (mm)
1	0.9–1.3	87.08	1.08
2	1.1–1.5	84.72	1.27
3	1.05–1.65	86.17	1.35
4	1.05–1.65	89.88	1.40
5	1.3–1.7	93.05	1.47
6	1.1–1.5	97.42	1.29
7	0.9–1.3	90.88	1.15
8	0.9–1.1	90.41	0.97
9	0.7–0.9	89.01	0.79

### Effect of Stirring Speed on the Macromorphology and Particle Size of PS/Fe_3_O_4_ Composite Beads

**FIGURE 6:**
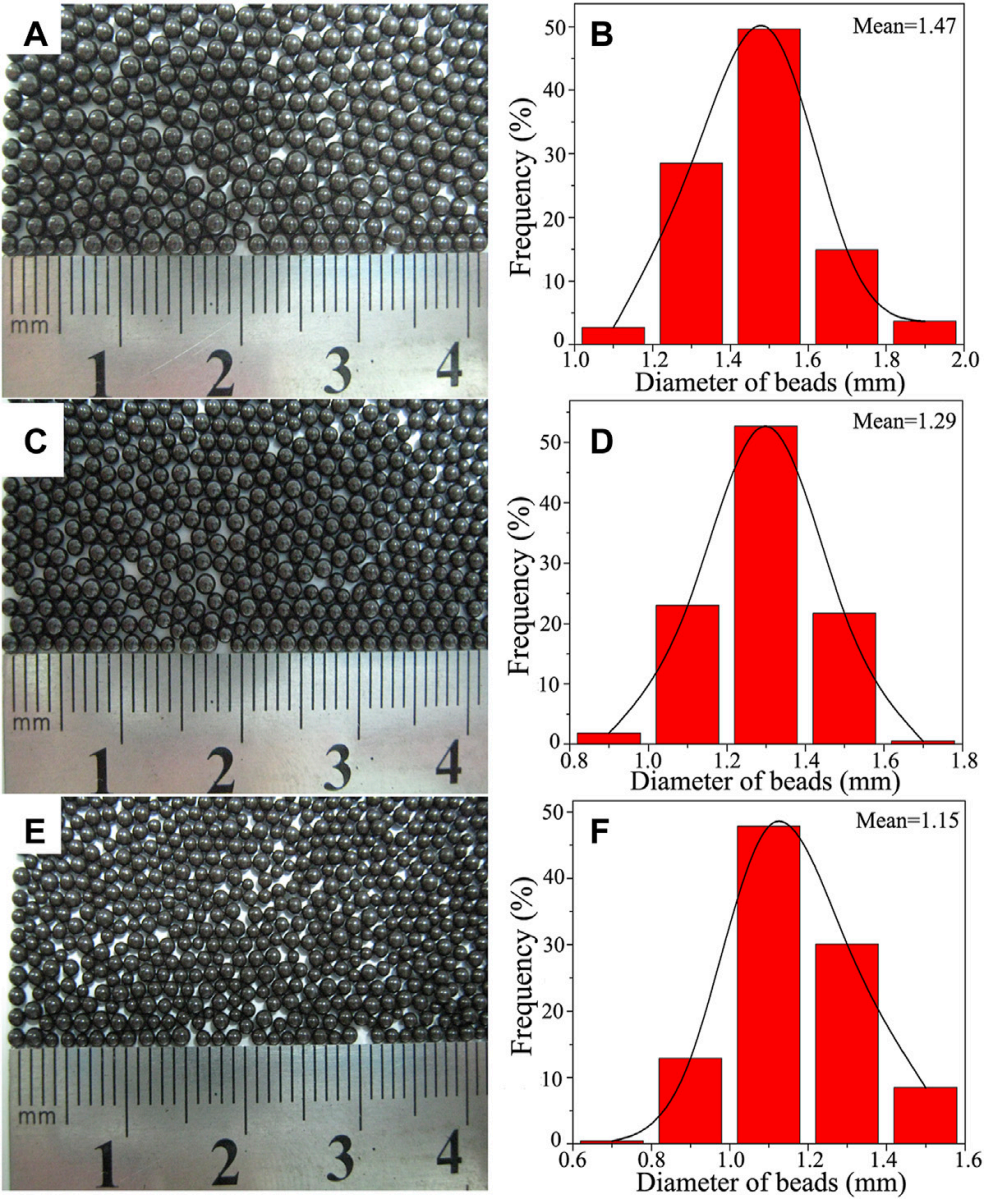
Optical photography showing the macromorphology of PS/Fe_3_O_4_ composite beads with the OA-Fe_3_O_4_ content of 2 wt% obtained at different stirring speeds: **(A)** 180 rpm, **(C)** 210 rpm, and **(E)** 240 rpm. Panels **(B)**, **(D),** and **(F)** are the histograms showing the size distribution of panels **(A)**, **(C),** and **(E)**, respectively.

It is known that the control of droplet coalescence and break-up rates are critical for the synthesis of polymer beads with uniform size. In order to realize the controllable synthesis of PS/Fe_3_O_4_ composite beads with desired particle size and narrow size distribution, the effect of stirring speed on the macromorphology and particle size of PS/Fe_3_O_4_ composite beads was examined, and a series of PS/Fe_3_O_4_ composite beads with an OA-Fe_3_O_4_ content of 2 wt% were synthesized at different stirring speeds according to the recipes in Table 1. As shown in Figure 6, the achieved PS/Fe_3_O_4_ composite beads were black with a smooth surface. In addition, the statistical histogram of the achieved PS/Fe_3_O_4_ composite beads is shown in Figure 6, and the detailed results are listed in Table 2, which vividly showed that the stirring speed could regulate the particle size and size distribution of PS/Fe_3_O_4_ composite beads. And the particle size distribution of the achieved PS/Fe_3_O_4_ beads in Figures 6A,C,E mainly concentrated in the range of 1.3–1.7, 1.1–1.5, and 0.9–1.3 mm, accounting for 93.05, 97.42, and 90.88%, respectively, and their respective mean diameters were 1.47, 1.29, and 1.15 mm. Based on the above analysis results, it could be concluded that the narrow size distribution was maintained, and the mean diameter decreased with increasing stirring speed; it is attributed to the increase in the stirring speed, which resulted in the increase in the polymerizing monomer droplet break-up rate and effectively prevented the monomer droplets from coalescence. Therefore, the better control of coalescence and break-up rates was therefore critical for the production of PS/Fe_3_O_4_ beads with uniform size.

### Effect of Sodium Dodecylbenzene Sulfonate Dosage on the Particle Size of PS/Fe_3_O_4_ Composite Beads

**FIGURE 7:**
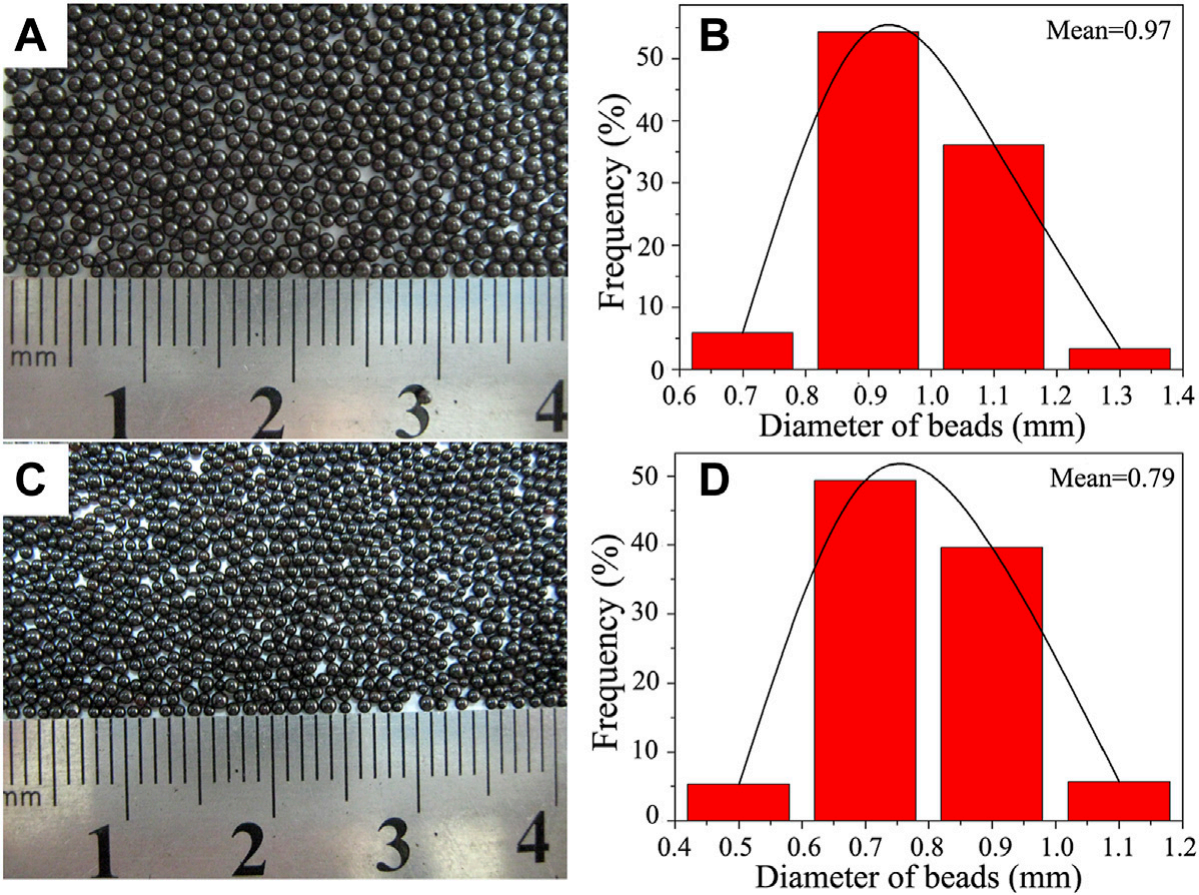
Optical photography showing the macromorphology of PS/Fe_3_O_4_ composite beads obtained with different dosages of SDBS aqueous solution: **(A)** 10 ml and **(C)** 20 ml. Panels **(B)** and **(D)** are the histograms showing the size distribution of panels **(A)** and **(C)**, respectively.

Extensive studies had demonstrated that the major issue in the suspension polymerization system was the formation of stable monomer droplets, preferably having a uniform particle size and a narrow particle size distribution. The mean size of the polymer beads was expected to be approximately the same as that of the initial monomer droplets. If the initial monomer droplet was monodisperse, then suspension polymerization would result in a relatively narrow particle size distribution. In order to further explore the effect of surfactant SDBS dosage on the stability of styrene monomer droplets, the PS/Fe_3_O_4_ beads of samples 8 and 9 were thus synthesized only by varying the SDBS dosage according to the recipe in Table 1. As shown in Figures 7A,C, the as-prepared PS/Fe_3_O_4_ composite beads possessed an excellent spherical profile, a smooth surface, a uniform size, and a black luster. As seen from Figures 7B,D, the particle size distribution of the achieved PS/Fe_3_O_4_ beads mainly concentrated in the range of 0.9–1.1 and 0.7–0.9 mm, accounting for 90.41 and 89.01%, respectively, and their respective mean diameters were 0.97 and 0.79 mm. It is suggested that the narrow size distribution was maintained, and the mean diameter decreased with the increase in the surfactant SDBS dosage; this is due to the fact that surfactant SDBS could improve the protective efficacy of the inorganic stabilizer TCP and control the contact angle, which played a critical role in controlling the adsorption of inorganic stabilizers at the oil/water interface. Therefore, the stability and size of initial styrene monomer droplets were highly dependent on the surfactant SDBS dosage. There is a synergistic effect between surfactant SDBS and the inorganic stabilizer TCP, which codetermined the controllable synthesis of PS/Fe_3_O_4_ composite beads.

### Fourier Transform Infrared Spectrum

**FIGURE 8:**
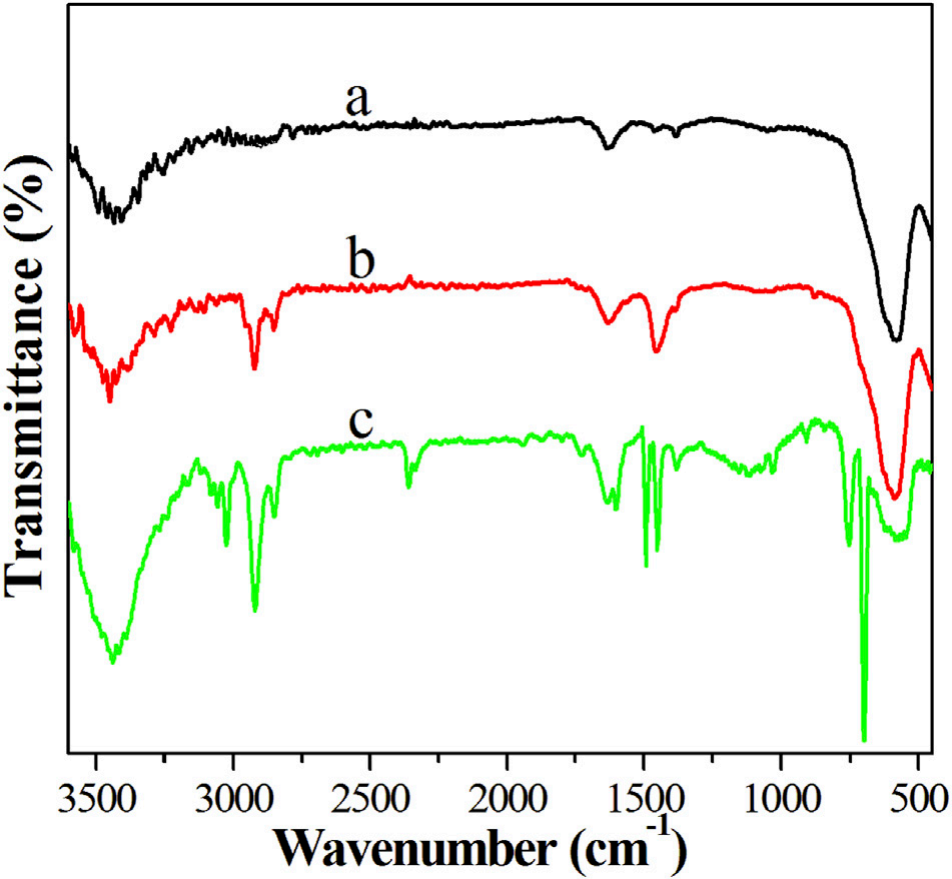
FT-IR spectrum: **(A)** Fe_3_O_4_, **(B)** OA-Fe_3_O_4_, and **(C)** PS/Fe_3_O_4_ composite beads.

Fe_3_O_4_, OA-Fe_3_O_4_ nanoparticles, and PS/Fe_3_O_4_ composite beads were analyzed by FT-IR spectrum in Figure 8. The peaks at 578 and 1631 cm^−1^ in Figure 8A were the Fe-O vibration and hydroxyl adsorption of Fe_3_O_4_ (Wang et al., 2013). The curve seen in Figure 8B of the OA-Fe_3_O_4_ nanoparticle exhibited not only the characteristic adsorption peaks of Fe_3_O_4_ (578 cm^−1^) and OA (2860 and 2930 cm^−1^) but also the characteristic vibration peaks of RO-Fe (1452 cm^−1^), and the results suggested that the OA molecule was successfully grafted on the Fe_3_O_4_ nanoparticles. Based on the curve (Figure 8C) of the PS/Fe_3_O_4_ composite bead, it was noted that there were intense specific adsorption peaks of PS (3065–2830, 1601–1350, and 700 cm^−1^), which were attributed to the stretching vibrations of the aromatic C–H in-plane, the stretching vibrations of the aromatic C–C, and the bending vibrations of the aromatic C–C out-of-plane, respectively (Wang et al., 2010; Yang et al., 2010); moreover, the characteristic adsorption peak of Fe_3_O_4_ (578 cm^−1^) was detected in Figure 8C, which further demonstrated that PS/Fe_3_O_4_ composite beads were successfully synthesized *via in situ* suspension polymerization.

### X-Ray Diffraction Pattern Analysis

**FIGURE 9:**
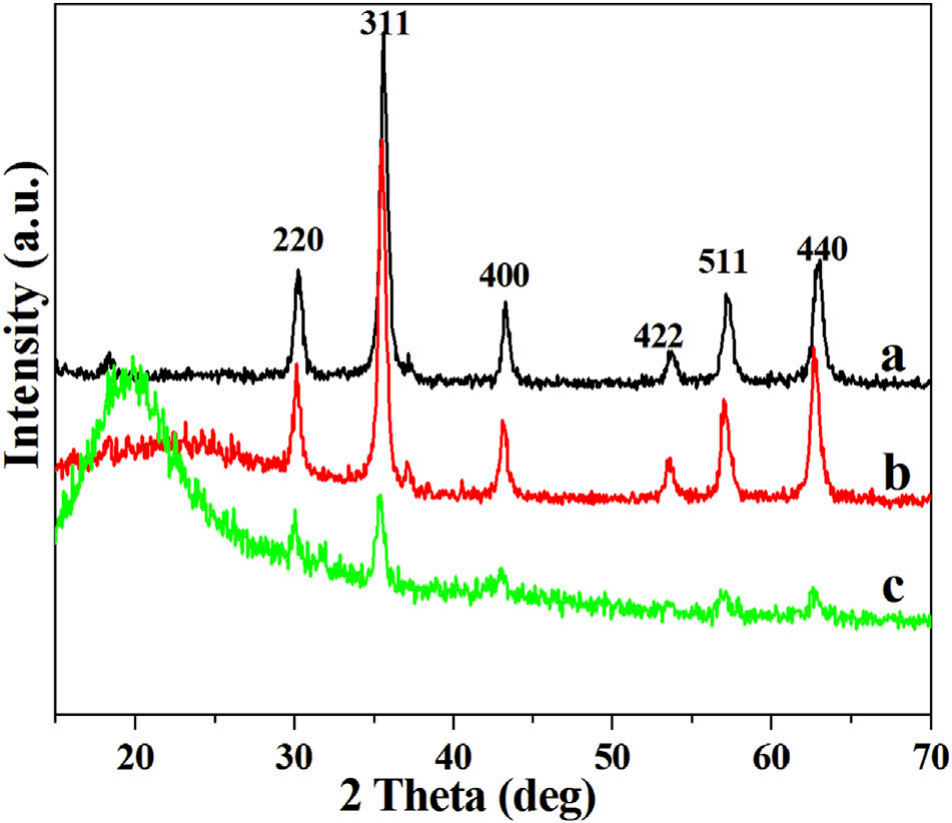
XRD patterns: **(A)** Fe_3_O_4_, **(B)** OA-Fe_3_O_4_, and **(C)** PS/Fe_3_O_4_ composite beads.

XRD patterns of Fe_3_O_4_ (a), OA-Fe_3_O_4_ (b), and PS/Fe_3_O_4_ (c) composite beads are shown in Figure 9. Based on Figure 9A, the characteristic diffraction peaks at 2 Theta of 30.2°, 35.7°, 43.3°, 53.6°, 57.2°, and 62.6° were assigned to (220), (311), (400), (422), (511), and (440) lattice planes of Fe_3_O_4_, respectively_._ It was clearly observed that XRD patterns in both Figures 9B,C possessed the same characteristic diffraction peak of Fe_3_O_4_, besides a broad peak at 2 Theta of 18–20° that corresponded to the amorphous peak of PS, which suggested that the crystal structure of Fe_3_O_4_ was not changed during the surface modification of Fe_3_O_4_ and *in situ* suspension polymerization of PS/Fe_3_O_4_. On the basis of the above analysis, it was confirmed that Fe_3_O_4_ nanoparticles were successfully incorporated into the PS matrix.

### Scanning Electron Microscopy, Energy-Dispersive Spectrometer, and Element Mapping Analysis

**FIGURE 10:**
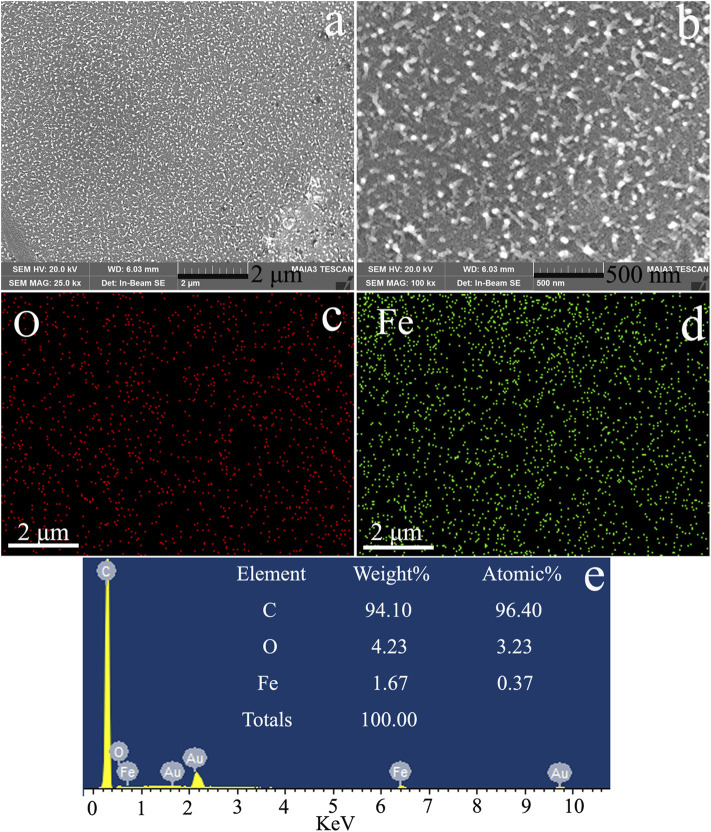
The cross-sectional morphology of PS/Fe_3_O_4_: **(A,B)** SEM images, **(C)** and **(D)** EM images of O and Fe, and **(E)** EDS image.

A key issue in the synthesis of PS/Fe_3_O_4_ composite beads was to disperse OA-Fe_3_O_4_ nanoparticles within the PS matrix homogeneously; thus, SEM, EDS, and EM were employed to observe the cross-sectional morphology of PS/Fe_3_O_4_ composite beads and the distribution of OA-Fe_3_O_4_ in the PS matrix. As seen from Figures 10A,B, it is noted that the Fe_3_O_4_ nanoparticles were well distributed in the PS matrix without obvious aggregation and phase separation phenomenon, and the mean size of Fe_3_O_4_ was about 95 nm, which was consistent with the DLS measurement result of OA-Fe_3_O_4_. The EDS of PS/Fe_3_O_4_ composite beads in Figure 10E displayed that besides C and Au peaks from the conductive tape, PS, and sprayed Au, only Fe and O were detected, which demonstrated that the Fe_3_O_4_ nanoparticles existed in the PS/Fe_3_O_4_ composite beads. For proving the distribution of Fe_3_O_4_ in the PS matrix, the EM of PS/Fe_3_O_4_ composite beads is examined in Figures 10C,D; the small plots denoted the distribution of each element in the PS/Fe_3_O_4_ matrix, and it is obvious that the characteristic Fe and O elements of Fe_3_O_4_ could be clearly observed in the EM of PS/Fe_3_O_4_ composite beads with a homogeneous distribution. Based on the above analysis, it is proved that the OA-Fe_3_O_4_ nanoparticles were successfully incorporated into PS beads and were well distributed in the PS matrix, and this phenomenon was attributed to the modification of Fe_3_O_4_ by OA molecules, which immensely improved the compatibility between the styrene monomer and Fe_3_O_4_ nanoparticles; it is demonstrated that the proposed method of *in situ* suspension polymerization was feasible for synthesizing the PS/Fe_3_O_4_ composite beads.

### Magnetic Property Testing

**FIGURE 11:**
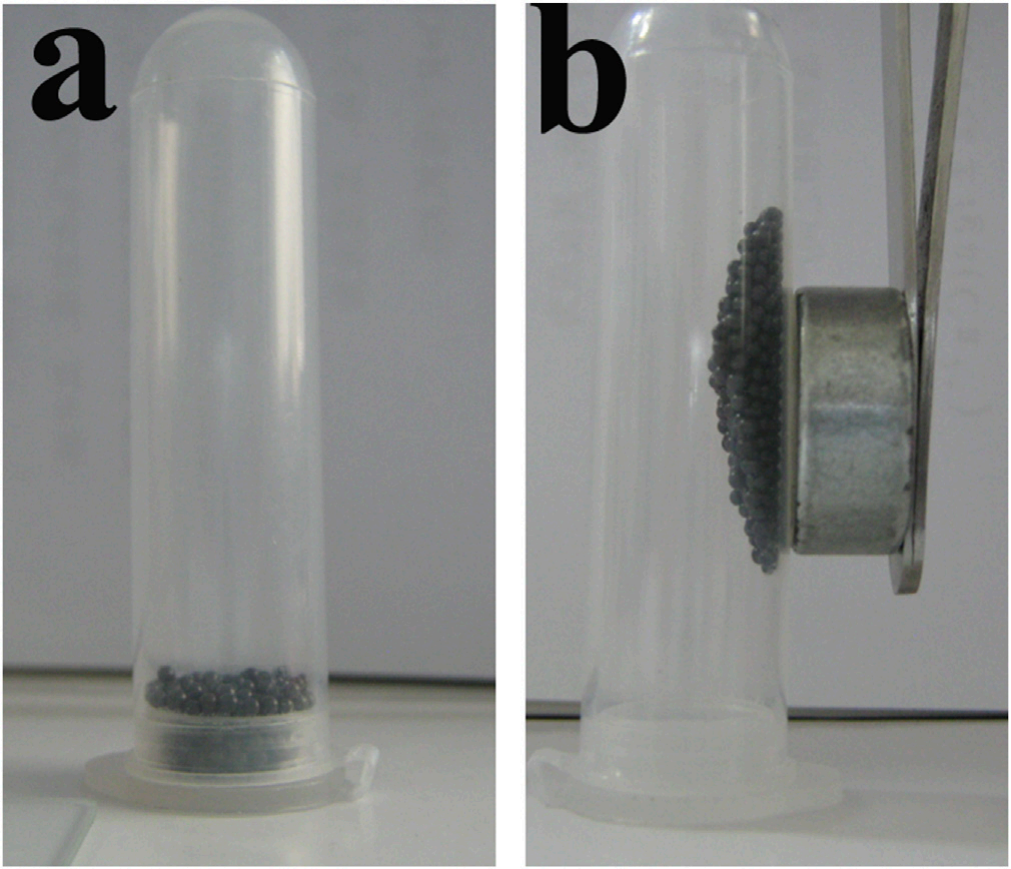
Optical photographs showing magnetic property: **(A)** PS/Fe_3_O_4_ beads and **(B)** the directed movement under an external magnetic field.

It is well known that the expanded PS foam particles were very easy to float due to their lightweight, static electricity, and airflow, which were very difficult to collect. The incorporation of Fe_3_O_4_ nanoparticles into the PS matrix not only imparted different color degrees to PS that could manufacture various color PS products, but also endowed with excellent magnetic property. Therefore, both the different color degree and excellent magnetic property of the PS/Fe_3_O_4_ composite beads could provide many functions for different products and applications, such as color PS beads, color-extruded PS board, color-expanded PS foam board, magnetic collection of PS/Fe_3_O_4_ composite beads or foam particles, product labeling for distinguishing different product classifications, and so on. As shown in Figure 11A, the PS/Fe_3_O_4_ composite beads without external magnetic fields were still at the bottom of the centrifuge tube. However, PS/Fe_3_O_4_ composite beads were easily collected by a magnet, and it could be concluded that the PS/Fe_3_O_4_ composite beads possessed excellent magnetic property.

### Thermal Stability Behaviors

**FIGURE 12:**
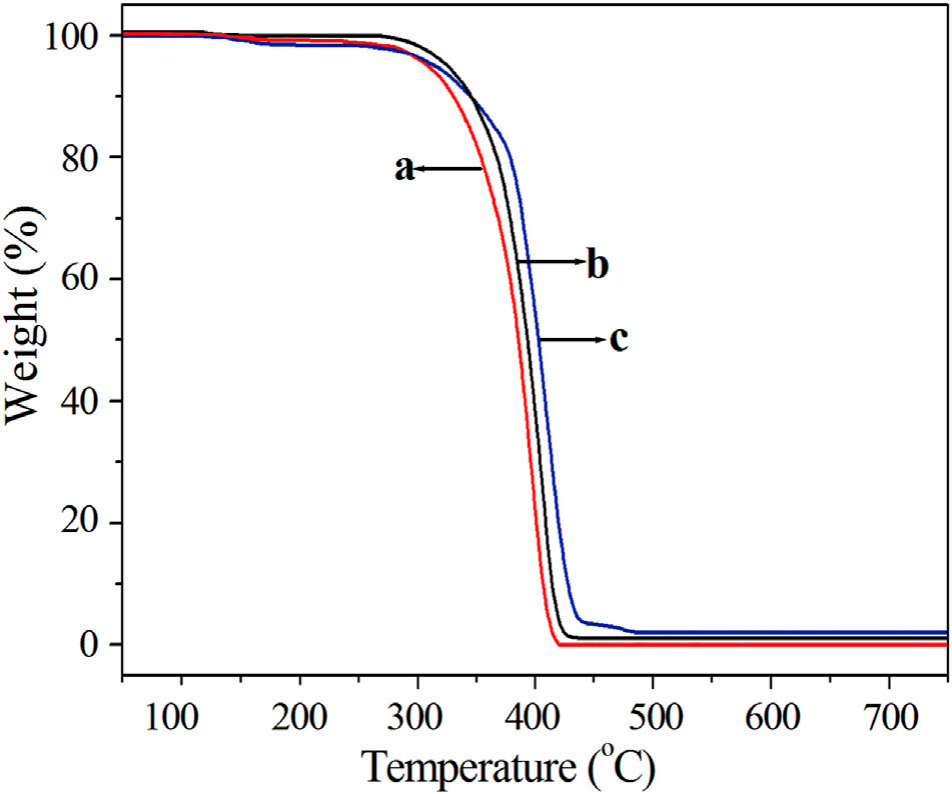
TGA curves: **(A)** PS, **(B)** PS/Fe_3_O_4_ with an Fe_3_O_4_ content of 0.8 wt% (sample 2), and **(C)** PS/Fe_3_O_4_ with an Fe_3_O_4_ content of 2 wt% (sample 5).

In order to investigate the effect of Fe_3_O_4_ dosage on the thermal stability of the resultant PS/Fe_3_O_4_ composite beads, TGA measurement was thus employed, and the TGA curves of pure PS, PS/Fe_3_O_4_ (0.8 wt%), and PS/Fe_3_O_4_ (2 wt%) composite beads are depicted in Figure 12. It could be seen that the thermal stability of pure PS, PS/Fe_3_O_4_ (0.8 wt%), and PS/Fe_3_O_4_ (2 wt%) was similar before 270°C. However, when the weight residual was 80%, the decomposition temperatures of curves (a)–(c) in Figure 12 were about 354.5, 368.5, and 379°C, respectively. When the weight residual was 50%, the decomposition temperatures of curves (a)–(c) were about 387, 393, and 402°C, respectively. On the basis of the above analysis, for the same weight residual, the decomposition temperature of PS/Fe_3_O_4_ composite beads was shifted toward higher temperatures with the increasing Fe_3_O_4_ content in comparison with pure PS beads, which demonstrated that PS/Fe_3_O_4_ composite beads possessed a better thermal stability than that of pure PS. This amelioration in the thermal stability of PS/Fe_3_O_4_ composite beads should be attributed to the incorporation of OA-Fe_3_O_4_ nanoparticles into the PS matrix, and this phenomenon could be interpreted from the following two aspects. On the one hand, OA-Fe_3_O_4_ nanoparticles possessed an excellent compatibility with styrene, so the polymerization of the styrene monomer took place on the surface of OA-Fe_3_O_4_ nanoparticles, and the physical crosslinking could be formed among the polystyrene chain segments, so the motion of the PS chain segment was confined due to the high packing density of PS chains around the Fe_3_O_4_ nanoparticles; on the other hand, the chemical bond might be formed between the polystyrene and Fe_3_O_4_ *via* the copolymerization of styrene and the double bond of OA molecular, so the Fe_3_O_4_ nanoparticles confined the movement of PS chain segments during the thermal degradation of polystyrene, both of which resulted in the improvement of thermal stability of PS/Fe_3_O_4_ composite beads. Similar improvement in the thermal stability of PS was observed when the modified Fe_2_O_3_ was incorporated into the PS matrix (Marinovic-Cincovic et al., 2006; Qiu et al., 2006; Qiu et al., 2007). Furthermore, the pure PS of curve (a) showed no residual weight due to complete decomposition; however, curves (b) and (c) exhibited weight residuals of about 0.76 and 1.97 wt%, respectively, which were almost identical with the original dosage; it is suggested that the Fe_3_O_4_ nanoparticles were successfully modified by the OA and fully incorporated into the PS matrix.

### Investigation on the Applicability and Simplicity of Polymerization Method

**FIGURE 13:**
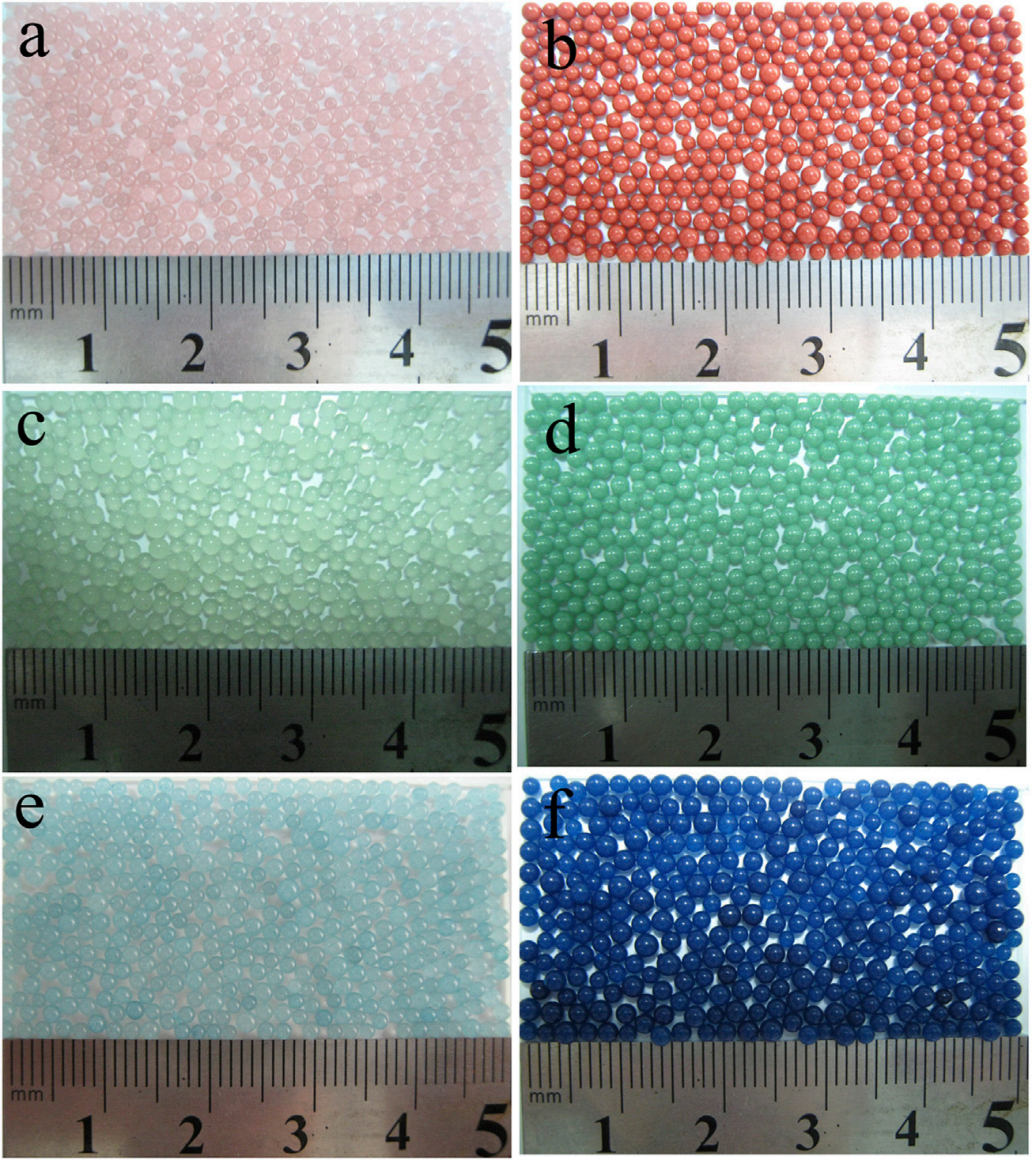
Colorized PS composite beads with different modified pigments: **(A,B)** iron oxide red, **(C,D)** dark green, and **(E,F)** China blue.

A series of colorized, magnetic, and millimeter-sized PS/Fe_3_O_4_ beads were successfully synthesized *via in situ* suspension polymerization of styrene. A versatile, scalable, and facile method was firstly reported. Three kinds of different inorganic pigments were modified according to the modification process of Fe_3_O_4_, and different colored PS beads in Figure 13 were successfully achieved *via in situ* suspension polymerization, which demonstrates that the modification process and polymerization process possess good versatility and applicability. Consequently, our proposed method can be applied for the industrial large-scale production of colorized PS and EPS beads, and this method may be readily extended to synthesize other classes of colored magnetic polymer hybrids based on the modified Fe_3_O_4_, such as polyvinyl chloride, polymethyl methacrylate beads, and so on.

## Conclusion

In this work, colorized and magnetic PS/Fe_3_O_4_ beads with millimeter size were first synthesized *via in situ* suspension polymerization of styrene. Effects of the modified Fe_3_O_4_ content, stirring speed, and surfactant dosage on the particle size of PS/Fe_3_O_4_ beads were systematically examined. The particle size, size distribution, and color degree can be easily regulated by changing the OA-Fe_3_O_4_ dosage. SEM and Fe and O EM results indicate that Fe_3_O_4_ is well dispersed in the PS/Fe_3_O_4_ bead. The proposed method can be applied for the industrial large-scale production of colorized PS and EPS beads. The process for synthesizing PS/Fe_3_O_4_ beads is extended to synthesize other three kinds of colorful PS beads, which demonstrate that our proposed process is versatile, scalable, and applicable to synthesize other classes of colorized and magnetic polymer hybrids based on the modified Fe_3_O_4_, such as polyvinyl chloride, polymethyl methacrylate beads, and so on.

## Data Availability

The raw data supporting the conclusions of this article will be made available by the authors without undue reservation.
